# Prayer-marks Heralding Acute Coronary Syndrome

**Published:** 2011-12

**Authors:** Vishal Sharma, Alka Sharma, Sourabh Aggarwal

**Affiliations:** Department of Medicine, University College of Medical Sciences, New Delhi 11095, India

Sir,

We read, with interest, the case report on extension of prayer-marks by Cangiano *et al.* and its association with worsening of the underlying chronic disease (1). We report a case where the occurrence of a similar skin lesion preceded an episode of acute coronary syndrome.

A 45-year old man presented with sudden onset of severe retrosternal chest pain radiating to the left arm, which was associated with sweating. His ECG revealed changes consistent with anterior wall myocardial infarction. Hyperpigmented areas over the middle of his forehead (Fig. 1) and knees (Fig. 2) had developed over the last two months. He was a Muslim by religion and regularly attended to his religious prayers (*Namaaj*), which involves kneeling and touching the ground with the forehead. Such skin changes, called *Namaaj* sign by us, have been described in earlier reports as a ‘prayer sign’ or ‘prayer nodules’ but no name has been assigned to the skin lesions (2,3). These lesions have been found to be associated with lichenification, acanthosis, basal cell hyperpigmentation, hyperkeratosis, hypergranulosis, and dermal papillary fibrosis (2).

**Fig. 1.**
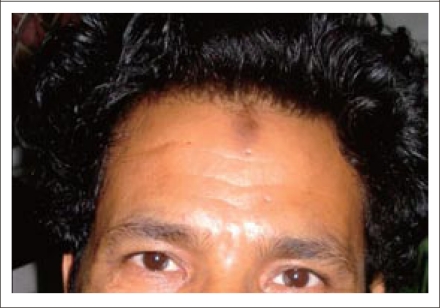
Hyperpigmented area over the middle of forehead

Our report emphasizes the association of the prayer sign (*Namaaj* sign) with acute conditions in addition to chronic conditions as suggested by Cangiano *et al.* However, further studies are needed to prove a statistically significant link between the two entities.

**Fig. 2.**
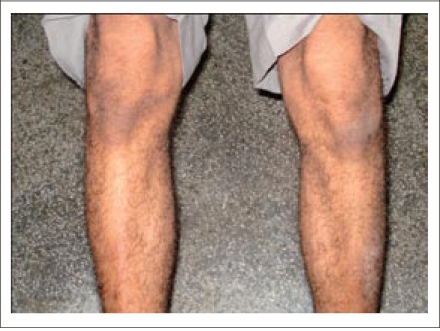
Hyperpigmented areas of knees

## ACKNOWLEDGEMENTS

The authors thank Dr. Shridhar Dwivedi for introducing the term *Namaaj* sign to them.
